# Nanostructured Lipid Carrier–Mediated Transdermal Delivery of Aceclofenac Hydrogel Present an Effective Therapeutic Approach for Inflammatory Diseases

**DOI:** 10.3389/fphar.2021.713616

**Published:** 2021-09-20

**Authors:** Neeraj K. Garg, Nikunj Tandel, Sanjay Kumar Bhadada, Rajeev K. Tyagi

**Affiliations:** ^1^ University Institute of Pharmaceutical Sciences, Panjab University, Chandigarh, India; ^2^ Institute of Science, Nirma University, Ahmedabad, India; ^3^ Department of Endocrinology, Postgraduate Institute of Medical Education and Research (PGIMER), Chandigarh, India; ^4^ Division of Cell Biology and Immunology, Biomedical Parasitology and Nano-Immunology Lab, CSIR-Institute of Microbial Technology (IMTECH), Chandigarh, India

**Keywords:** aceclofenac, nanostructured lipid carrier, rheological behavior, texture profile, transdermal delivery

## Abstract

Aceclofenac (ACE), a cyclooxygenase-2 inhibitor, is the derivative of the diclofenac group that has been in use for the symptomatic treatment of systemic inflammatory autoimmune disease, rheumatoid arthritis (RA). Partial solubility, high lipophilic nature, and stability challenge its use in developing topical formulations. Hence, we developed and characterized nanostructured lipid carrier (NLC)–based ACE (ACE-NLC) hydrogel for an efficient transdermal delivery. NLC microemulsion was prepared using different lipids by various methods and was characterized with respect to particle size, zeta potential, surface morphology, and drug encapsulation efficiency. The optimized NLC formulation was incorporated into Carbopol^®^ 940 gel, and this arrangement was characterized and compared with the existing marketed gel (Mkt-gel) formulation to assess *in vitro* drug release, rheology, texture profile, *in vivo* skin retention and permeation, and stability. Furthermore, prepared and characterized ACE-loaded NLC formulation was evaluated for skin integrity and fitted in a dermatokinetic model. The results of this study confirmed the spherical shape; smooth morphology and nanometric size attested by Zetasizer and scanning and transmission electron microcopy; and stability of the ACE-NLC formulation. The ACE-NLC-gel formulation showed good rheological and texture characteristics, and better skin distribution in the epidermis and dermis. Moreover, ACE-NLC permeated deeper in the skin layers and kept the skin integrity intact. Overall, NLC-based gel formulation of ACE might be a promising nanoscale lipid carrier for topical application when compared with the conventional Mkt-gel formulation.

## Introduction

Nanostructured lipid carriers (NLCs) are known impulsive colloidal nanoscale drug carriers for efficient transdermal delivery of drugs and are advantageous than other delivery systems for local topical applications (Müller et al., 2007; Garg et al., 2016b; Garg et al., 2016c; Chauhan et al., 2020; Haider et al., 2020; Kanojia et al., 2021). Colloidal nanoscale drug carriers (NLCs) render the controlled release profile of drugs and biologics (Souto et al., 2004; Souto and Müller, 2005; Scioli Montoto et al., 2020; Patil et al., 2021) to overcome the limitations observed with solid lipid nanoparticles (SLNs) (Khosa et al., 2018) that include suboptimal drug loading and gelation, and lipid polymorphism–associated leakage of drug upon storage (Garg et al., 2016a). NLCs give an occlusive effect and thus increase skin hydration which, in turn, enhances bioavailability of active compounds in the skin. Increased biodistribution and bioavailability confer greater physical stability of topical formulations (Chauhan et al., 2020). Since long-term oral use of aceclofenac (ACE) causes many side effects, including gastrointestinal ulcers and bleeding, ulcers of mucosal surface, stomatitis, suppression of the bone marrow, and drug-induced hepatic complications such as fibrosis and cirrhosis (Allison et al., 1992), the topical application of NLC-based ACE (ACE-NLC) formulation would be a preferred choice to treat inflammatory diseases. Consistent to what was reported earlier (Zweers et al., 2011), the NLC formulation comprised a mixture of solid lipid (long chain) and liquid lipid (short chain) at a preferred ratio from 70:30 to 99.9:0.1 (Patel et al., 2012). The matrix of the lipid particles exhibit a melting point depression compared to that seen with solid lipids, maintaining solidity of the matrix at an ambient temperature. The increased drug loading efficiency showed reduction in the expulsion of active compounds upon storage, and addresses the issue of reduction during the hydration of particulate suspension (Pardeike et al., 2009; Naseri et al., 2015). Considering all issues and advantages, NLCs were prepared and characterized using physiological and biodegradable lipids with minimal cell toxicity (Müller et al., 2007; Khosa et al., 2018; Chauhan et al., 2020). These nano-sized lipid particles ensure close proximity to the stratum corneum for increased penetration of the drug across the skin layers (dermis and epidermis) (Khater et al., 2021). The higher loading and entrapment efficiency (EE), protection rendered for entrapped drugs, minimum leakage of drugs due to lipid polymorphism and improved biocompatibility, *in vivo* stability of the drug formulation, better drug penetration and permeation across the skin layers, and relatively lesser systemic toxicity suggest topical delivery of ACE as a viable delivery approach to address the existing limitations (Raza et al., 2014). ACE, a nonsteroidal anti-inflammatory drug, is used to lessen rheumatoid arthritis (RA)–induced chronic inflammation and to alleviate pain in RA, osteoarthritis, and ankylosing spondylitis pathogenesis (Brogden and Wiseman, 1996; Arslan and Tirnaksiz, 2010). ACE, a prodrug of diclofenac, exhibits a multifactor mechanism underlying the action mediated by selective inhibition of prostaglandin E2 (PgE2) (Arslan and Tirnaksiz, 2010) and exerts anti-inflammatory effects on inflammation markers, including IL-1β, IL-6, and TNF-α (Syggelos et al., 2007). The anti-inflammatory effect of ACE is primarily due to the inhibition of cyclooxygenase that reduces the production of prostaglandins responsible for inflammation and joint pain (Zweers et al., 2011; Gunaydin and Bilge, 2018).

The oral administration and adverse effects rendered by ACE prompted researchers to formulate nanoscale drug carrier–mediated transdermal delivery for sustained delivery, to circumvent its adverse effects (Raza et al., 2014). Transdermal delivery of ACE has shown sustained release of drugs and biologics with better patient compliance (Alam et al., 2020) and induces the PI3-Akt signaling pathway to control apoptosis and immune effectors (manuscript under review). Transdermal route addresses gastrointestinal tract–associated adverse effects, increases patient compliance, and avoids first-pass metabolism, thereby increasing interest in using nanoscale drug carrier–loaded drugs through the topical route which modifies drug permeation through the skin (Modi and Patel, 2011; Garg et al., 2016b; Chaudhari et al., 2021). Stability of drug-loaded formulation, frequent dosing schedules, and difficult manufacturing processes raise the need for developing topical formulations than oral ACE formulations. Recently, lipid-based nanocarriers have emerged as a promising tool for transdermal delivery through different methods, such as nanoemulsions (Modi and Patel, 2011), SLNs (Jain et al., 2014), and NLCs (Hung et al., 2011) *via* engineering, the precision of which is based on nanotechnological advances (Chawla and Saraf, 2012) for drug delivery to enhance personalized interventions (Carbone et al., 2013; Yu Z. et al., 2021; Mitchell et al., 2021).

Garg et al. (2016b, 2016c) published *in vitro* and *in vivo* findings using NLC-mediated delivery of methotrexate that showed a higher expression of the proapoptotic gene, Bim, controlled by nuclear factor-κB (NF-κB) and forkhead box O1 protein (FOXO1), which is supported by the relative mRNA expression (fold regulations) of apoptotic and pro-inflammatory mediators in human U937 and experimental RA models. The findings of these studies suggested a reduction in inflammation and triggered programed cell death regulated by NF-κB and FOXO1 that were expressed following MTX-loaded NLC treatment in RA. In addition, formulated NLCs exhibited better skin permeation with higher permeation flux and enhancement ratio (ER) shown by confocal laser scanning microscopy.

This work presents the development of a new method for NLC formulations that improves the chemical stability of ACE and physical stability of ACE-NLCs. Solubility of ACE optimized in different solvents using combinations and permutations of surfactants and cosurfactants to prepare ACE-NLCs and the rheological behavior of NLC gel were assessed. Characterized ACE gel was evaluated for skin permeation (*ex vivo*), drug distribution, and various dermatokinetic parameters and compared with the marketed gel (Mkt-gel) formulation. Histopathology was carried out to detect integrity of drug-treated skin. In a nutshell, developed NLC formulations are efficacious for the transdermal delivery of ACE when compared with the Mkt-gel formulation. Overall, success associated with the use of ACE entrapped in NLCs would be mirrored to enhancing permeation of drugs across the skin layers because of the noninvasive regime of topical formulations of drugs and therapeutic biologics.

## Materials and Methods

### Materials

ACE was supplied as a gift sample from Ipca Laboratories, Mumbai, India. Lipids such as glyceryl monostearate (GMS), stearic acid (SA), and cetyl alcohol (CA) were purchased from Loba Chemie Pvt Ltd., Mumbai, India. Mkt-gel (1.5%) of ACE was procured from a local medical store. Liquid lipid Transcutol, Labrafac, and Labrasol were supplied by Gattefossé, France. Phospholipon S 100 was supplied as a gift sample by Sasol, Germany. Poloxamer (Pluronic F-68) was obtained as a gift sample from BASF, Mumbai, India. All other chemicals and reagents were of analytical grade, and solvents used for high-performance liquid chromatography (HPLC) were of HPLC grade unless otherwise specified.

### Solubility Studies

Solubility of ACE was studied in different oils, lipids, surfactants, and cosurfactants. Briefly, excess quantity of the drug was added individually into 1 ml of each oil, surfactant, and cosurfactant. Each sample was centrifuged, and 0.5 ml of the clear supernatant was diluted with methanol and then analyzed using HPLC. For the solubility of the drug in solid lipid, 1 g of various lipids was melted and the drug added individually in 50-mg increments with each addition. Tubes were continuously vortexed after each addition for faster dissolution of the drug. This procedure was continued till the molten lipid solubilizes the drug. Upon reaching the saturation level, tubes were vortexed, centrifuged, and the upper layer transformed in dissolved chloroform (CHCl_3_). Mobile phase diluted CHCl_3_ solution and drug were quantified by HPLC.

### Pseudoternary Phase Diagram

The microemulsion (ME) region was drawn by the heated lipid phase to melt the solid lipid, followed by heating and addition of ethanolic solution of phospholipid. The surfactant phase (S_mix_) and lipid phase were heated at the same temperature. At 60°C and normal room pressure, a mixture of a known amount of S_mix_ and lipid at various ratios was chosen and subsequently titrated with the aqueous phase (aqueous phase titration) with the help of a microsyringe. The solvent was mixed by gentle stirring every time, and then placed in a temperature-controlled water bath, and titrated until the solvent became turbid. Weight percent was calculated and plotted in Gibbs phase triangle as the boundary points after the final weight of the titrant at a particular S_mix_ of oil or aqueous ratio was determined, and the entire phase diagram was mapped by oil titration wherein the oil acted as the titrant (Garg et al., 2017).

### Preparation and Characterization of NLC

NLCs were prepared by the ME method using the ME region from the pseudoternary phase diagram. Three different equipment and protocols were used for NLC preparation to maximize the EE and to minimize drug degradation. NLCs prepared by these methods were named NLC-1, NLC-2, and NLC-3. The lipid phase comprised of solid lipid, oil lipid (Transcutol), and phospholipid in ethanol, whereas the aqueous phase comprised of water containing tween 80. Phospholipid was dissolved in ethanol and ACE in Transcutol. NLC-1 formulation was prepared by using high shear homogenizer (SilentCrusher M, Heidolph Instruments, Germany), and solid lipids (GMS/CA/SA) were added with Phospholipon solution in ethanol and heated at 55–60°C, then heated lipid oil (Transcutol) was added. After mixing the hot lipid phase, hot water was added to the lipid mixture. Primary ME was formed following stirring for 5 min, and the above prepared mixture was added to 0.5% poloxamer solution under homogenization. Thereafter, the suspension was homogenized at 8,000 rpm for 15 min by using high shear homogenizer followed by magnetic stirring for 2–3 h at 500 rpm. NLC-2 was prepared by using probe sonicator (Sonicator 3000, Misonix, Inc., NY, United States), and sonication applied for only primary ME for 3 min at 3–6 w. This suspension was then poured into cold water containing 0.5% poloxamer. The formulation was stirred for 2–3 h at 500 rpm, and NLC-3 was prepared by mixing, and probe sonication used for 30 s at 3 w for preparing the primary emulsion followed by the addition of drops in cold water (0.5% w/v poloxamer) with continuous homogenization at 5,000 rpm for 5 min. This prepared sample was stirred for 2–3 h at room temperature. α-Tocopherol acetate (0.05%) and propyl paraben (0.02%) were used as stabilizer and preservative, respectively. All excipients were added into lipid phase, and the formulations were prepared in dark, then dispersion of NLCs was dialyzed using the cellulose dialysis bag (MWCO; 10 kDa) against double distilled water and acetone mixture (2:1) to remove unentrapped or free drug. Excess lipid was removed by membrane filter (0.45 μm), Millipore, United States, and washed with deionized water. Separated NLC suspensions were lyophilized using VirTis AdVantage and stored for long term use.

#### Characterization of NLC

##### Size, Polydispersity Index, and Zeta Potential

The size and polydispersity index (PDI) of NLCs were determined using Zetasizer (PCS, Nano ZS90, Malvern Instruments, United Kingdom). Samples were prepared by diluting the final NLC suspension 10–15 times using deionized water kept in polystyrene cuvettes and observed at a fixed angle of 90° at 25 ± 0.1°C. The zeta potential of the formulated NLCs was determined in folded capillary cells by laser Doppler anemometry using Malvern Zetasizer. Measurements were carried out at 25 ± 0.1°C with the samples properly dispersed in deionized water kept in the electrophoretic cell at an electric field of 15.24 V/cm to determine the zeta potential (Eratte et al., 2014).

##### Surface Morphology

The surface morphology of the prepared NLC formulation was confirmed by transmission electron microscopy (TEM) and is in accordance with the findings of Garg et al. (2016c, 2017) and Chauhan et al. (2020). Scanning electron microscopy (SEM) analysis provides high-resolution imaging useful to determine surface fractures, flaws, contaminants, or corrosion. A drop of diluted NLC suspension was taken on a coverslip which was fixed on to a brass stub using a double-sided adhesive tape and analyzed using SEM. It was then dried and made electrically conducive by coating it with a thin layer of gold; the sample was then loaded onto the microscope to take images. TEM analysis was conducted by placing a drop of diluted sample on a membrane-coated grid surface and further stained using a drop of 1% phosphotungstic acid and immediately added to the grid surface for 1 min, then the excess fluid was removed and the grid surface air dried at room temperature. The sample was then examined using high-resolution (HR)–TEM, Tecnai 200 Kv TEM at 10 × 15,000 magnification (Garg et al., 2016c; Garg et al., 2017; Chauhan et al., 2020).

##### Drug Encapsulation Efficiency

The drug encapsulation efficiency was determined by using the direct lysis method as reported by Garg et al. (2017). Briefly, ultracentrifugation was performed at 40,000 rpm for 20 min at 4°C using 20 ml solution of NLCs, and pellets were washed three times using double-distilled water to separate the unentrapped drug. Next, the pellets were lysed using chloroform diluted with methanol followed by filtration through 0.22-mm filters (Millipore, United States). The drug was quantified from the filtrate using the HPLC method (Sharma et al., 2016a). The HPLC system (Shimadzu LC-2010C HT ver. 3.01 system, M/s Shimadzu Inc., Tokyo, Japan) was connected with a UV-vis detector (equipped with a quaternary pump, mobile phase degasser, column thermostat controller, and SPD-10AVP column oven). C18 columns from Thermo Fisher Scientific Inc., United States, were used for the separation. Isocratic elution (methanol:water, 70:30% v/v having 0.02% orthophosphoric acid) was used as the mobile phase at 275 nm. The flow rate was kept at 1 ml/min at room temperature for 20–30 μl of injection volume.

$$\text{Entrapment Efficiency }\left(\text{\%}\right)=\frac{\text{Amount of drug entrapped in NLCs}}{\text{Total amount of drug added}}\times 100.$$

##### Quantification of Drug by HPLC

The drug content was quantified by using the HPLC system (Shimadzu, Japan), equipped with a double reciprocating pump and photodiode array detector, operating at 254 nm, and with a Thermo Hypersil BDS C18 column (250 mm × 4.6 mm, 5 µ), Thermo Scientific, United States. The sample was analyzed by the isocratic elution mode with mobile phase, methanol: 0.02% orthophosphoric acid at 70:30 v/v ratio. The injection volume was kept at 20–30 µl with a flow rate of 1 ml/min at room temperature (Garg et al., 2016c).

### Preparation and Characterization of NLC Gel

#### Preparation of NLC-Incorporated Carbopol Hydrogel

Optimized NLC-3 dispersions were separately incorporated into concentrated Carbopol^®^ 940 gel base in accordance with the procedure described by Jain et al. (2014) and Garg et al. (2016b, 2016c) to obtain appropriate viscosity for the transdermal application. In brief, prepared dispersion of ACE-NLCs along with the chemical enhancer [α-terpineol 2% v/v in 70% isopropyl alcohol as 80:20 (ACE-NLC:CE)] was incorporated in the concentrated Carbopol 940 gel. NLC-incorporated gel was formulated, and the final drug concentration kept at 1.5%, and then evaluated with respect to color, grittiness, esthetic appeal, rheological behavior, and texture analysis.

#### Rheological Behavior

Rheology and texture analysis of the formulations were made following the protocols published by Jain et al. (2014), Souto et al. (2005), and Garg et al. (2016b, 2016c). Rheological behavior of NCL gel was determined using a dynamic rheometer (Anton Paar, Germany) equipped with a cone and plate geometry (cone diameter, 75 mm; cone angle, 0.999°) at 25°C, as reported previously by Garg et al. (2016c). Samples were charged on the plate and parameters adjusted as per the recommendations provided for the equipment. Flow curve test was conducted to determine the flow behavior of samples under changing shear rate (γ in s^−1^, range from 0.1 to 100 s^−1^) as a function of apparent viscosity (Pa^.^s) and shear stress (τ). The data obtained were processed and analyzed using the instrument software RheoPlus, and the linear viscoelastic region (LVR) was determined using the amplitude sweep test by measuring G′ (storage modulus) and G″ (loss modulus) as a function of strain (%) ranging from 0.01 to 100% at a constant angular frequency of 1 and an angular velocity of 10 rad/sec (Chen et al., 2012; Jain et al., 2014; Romero De Avila et al., 2014; Mahmood et al., 2021). The LVR provides information on the minimum strain required for the oscillation frequency sweep test that was carried out by measuring G′ and G″ as a function of angular frequency (rad/sec) at 0.1–100 rad/sec and a constant strain amplitude of 0.05 and 5% in the LVR (Mahmood et al., 2021). Following the above-mentioned protocol, a test was carried out to monitor sample behavior at constant strain and changing frequency.

#### Texture Profile Analysis

Texture profile analysis (TPA) was carried out using TA.XTplus Texture Analyzer (Stable Micro Systems, United Kingdom) to study stickiness and firmness of the prepared NLC gel. Upper (male) *vs.* lower (female) cones were calibrated before testing, and similar test conditions to that reported by Garg et al. (2016b, 2016c) were followed. Before testing, the upper cone (male cone) probe was calibrated against the lower cone (female) to adjust the height to about 25 mm above the lower cone. The equipment was equilibrated and maintained at 32°C, and the recommended volume (5 g) of NLC gel placed on the lower stage of the equipment to note down readings on samples. Force encountered by the male cone to break away from the gel when starting to ascend (the point of maximum force) was measured. The formulation was added to the female cone with utmost care to avoid air entrapment, and excess formulation was removed using the Exponent 32™ software (Jain et al., 2014; Negi et al., 2014; Madan et al., 2020). Furthermore, the recommended volume of the NLC gel was charged over the lower stage of the equipment, and readings were noted down. The texture profiling was assayed by directing the male cone to penetrate and detach from the test formulation present in the female cone, and the force applied by the male cone to break away from the gel was measured as mentioned by Garg et al. (2016c, 2017). The value of the peak force/measurement of the gel strength and area of curve (measurement of work of shear) was also measured. Higher the value of peak force higher the gel strength, as peak force is directly proportional to gel strength (Jones et al., 1996).

#### *In Vitro* Drug Release

Dialysis tube diffusion technique was used to assess *in vitro* drug release profiling of the entrapped drug from NLCs and NLC-gel formulations. NLC dispersion free from the unentrapped drug and the weighed amount of NLC gel with an equivalent quantity of drug were kept individually in a dialysis membrane (MWCO 10–12 kDa; Sigma, United States). The bag was tied at both ends and placed in a container filled with 30–40 ml of the solvent mixture with 70:30 (v/v) phosphate buffer saline (PBS) and ethanol (Jain et al., 2014; Garg et al., 2016c). Containers were assembled above a magnetic stirrer to achieve continuous stirring at 500–750 rpm and maintained at a constant temperature 32 ± 0.5°C. 1 ml of the sample was withdrawn intermittently from zero to 45 min and 1 to 48 h. The withdrawn sample was replaced with an equal volume of the solvent mixture in the receptor compartment as reported elsewhere (Zur Mühlen et al., 1998). Samples were analyzed to quantify ACE by the HPLC method as described in the *Characterization of NLC* section. Data were fitted into various drug release kinetic models, to name a few: zero order, first order, Higuchi model, Hixon–Crowell model, and Korsmeyer–Peppas model (Jain et al., 2014). The model that best fit the release data was evaluated by correlation coefficient (r) value. The correlation coefficient (r) values were used as a criterion to choose the best model to describe drug release from the NLC formulations. Regression analysis was performed to draw a graph of the models according to the need of each and every equation (Costa and Sousa Lobo, 2001; Mircioiu et al., 2019; Wu et al., 2019).

### *Ex Vivo* Skin Permeation Assays

Permeation study was conducted on the shaven skin of the ear pinnae of a pig procured from a slaughter house, Chandigarh, India. Hairs on the skin were removed carefully with the help of a surgical blade, and the skin was washed and properly rinsed with saline. Any fat material found adhered to the skin was wiped 3 to 4 times with isopropanol-soaked cotton swabs and detached from the dermis. Before use, these tissue samples were stored at −20°C for a maximum of 1–2 weeks (Kumar et al., 2007).

Skin permeation studies were performed on a well-jacketed Franz diffusion cell assembly (PermeGear, Inc., PA, United States). The jacketed cell comprised a donor compartment, receptor compartment, and a sampling port. The area of the donor compartment (diffusion cross-sectional area was 3.142 cm^2^) was exposed to the receptor compartment with a total capacity of 30 ml. The receptor medium was continuously stirred all throughout the study using an in-built magnetic stirrer. The temperature of the receptor medium was maintained at 32 ± 0.5°C by circulating warm water in the outer jacket of the diffusion cells using a thermostatically controlled water circulator. The harvested skin section was mounted on the diffusion cell with the stratum corneum surface facing up, while the donor compartment was kept dried and open to the atmosphere. The skin (thickness 1–1.5 mm) was clamped on a Franz diffusion cell in such a manner that the stratum corneum side faced upward toward the donor compartment and the dermal side faced downward towards the receptor compartment. The receiver compartment was filled with PBS (pH 7.4): absolute ethanol (7:3) and stirred continuously at 500 rpm. The skin tissue was allowed to equilibrate with the sink medium to maintain a skin hydration gradient (Warner et al., 1988) and generate the driving force for skin penetration of deformable vesicles (Cevc and Blume, 1992). The receiver content was then replaced by fresh medium.

As described by Garg et al. (2016c, 2017), marketed gel (Mkt-gel), NLCs, and NLC gel (1.8 mg/cm^2^, i.e., ∼5620 µg in each cell) were applied gently in the donor compartment, and 0.2 ml of the sample from the receiver compartment was drawn periodically (0.5 through 24 h), and an equal volume of PBS: ethanol solution was added to the receptor compartment to keep the sample size constant (Mei et al., 2003). Samples were filtered through an aqueous 0.22-µm pore size cellulose membrane filter and cumulative volumes of the drug permeated through the skin were analyzed (Jain et al., 2014). The cumulative amount of ACE was calculated (Negi et al., 2014). At the end point (i.e., 24 h), the donor compartment and the skin surface(s) were washed four to five times with the receptor medium. The obtained samples were diluted with suitable solvents and quantified for drug(s) using HPLC method(s), as described earlier. In the end, the raw data obtained with the diffusion drug release studies were analyzed by applying correction factor for the volume and drug losses during sampling by the replacement method (Singh et al., 2011; Garg et al., 2016c; Weng et al., 2020).

### *Ex Vivo* Skin Distribution Study

For the skin distribution study, the skin was removed, and the formulation was scrapped off by a scraper to get most of the adhered cells. The skin tissue was washed three times with deionized water and the epidermal and dermal layers manually separated using tweezers. These skin layers were chopped off to pieces and homogenized in 7–10 ml of methanol for 8–10 h for complete extraction of the drug (Kilfoyle et al., 2012; Raza et al., 2013; Jain et al., 2014). The extracted drug was filtered through a 0.22-µm membrane filter, and the filtrate was further analyzed to quantitate ACE.

The ER was calculated for each NLC-gel formulation and compared with that of the existing Mkt-gel formulation. The ER is defined as the ratio of % increase in the permeation parameter of the NLC-gel formulation to the amount of permeated Mkt-gel formulation (Raza et al., 2013; Jain et al., 2014; Garg et al., 2016c).

$$\mathrm{Enhancement}\;\mathrm{ratio}\;=\;\frac{\mathrm{Permeation}\;\mathrm{parameter}\;\mathrm{of}\;\mathrm{NLC}\;\mathrm{gel}\;\mathrm{based formulation}}{\mathrm{Permeation parameter of Marketed gel formulation}}.$$

### Dermatokinetic Modeling

Dermatokinetic modeling was carried out as reported elsewhere (Raza et al., 2013; Thotakura et al., 2017; Rapalli and Singhvi, 2021), where the ear pinnae of a pig was used for carrying out *ex vivo* studies. Franz diffusion cell assembly was used for the studies as discussed under the *Ex Vivo* Skin Permeation Assays section. The whole skin was removed from the Franz cell at determined sampling times for 24 h. The obtained data were fitted into a one-compartment open model, and different dermatokinetic parameters were calculated using the following formula (Raza et al., 2013; Garg et al., 2016c). The quantity of the drug in the epidermis and dermis was determined, and the obtained data were fitted in the one-compartment open model using the following formula:

$$C_{skin}=\frac{K_{p}.C_{max}^{skin}}{\left(K_{p}-K_{e}\right)}\left(e^{-K_{p}t}-e^{-k_{e}t}\right).$$

Here, $C_{Skin}$ : drug concentration in the skin at time *t*, *K*_*p*_: dermal permeation constant, $C_{Max}^{skin}$ : maximum concentration achieved in the skin, and *K*_*e*_ is the skin elimination constant. WinNonlin Ver 5.0 software was used to compute various dermatokinetic parameters: *K*_*p*_, $C_{Max}^{skin}$ , *K*_*e*,_ $T_{Max}^{skin}$ (time required to achieve $C_{Max}^{skin}$ ), and the area under the curve (AUC_0−24hrs_) using the Wagner−Nelson method.

The obtained data were analyzed using minimization of nonlinear function by the Gauss theorem algorithms built into the software. In accordance with the findings of Raza et al. (2013), the results obtained in this study were confirmed using the minimization of various model fitness parameters, such as the Akaike information criterion, Schwartz criterion, sum of squares due to residuals, and maximization of the Pearson correlation coefficient.

### Assessment of the Skin Integrity by Histopathology

Histopathological assays were carried out to assess skin integrity. The skin tissues were cut into pieces, fixed in 10% buffered formalin, embedded in paraffin, and 5-µm sections prepared using a microtome. The skin sections were stained using hematoxylin and eosin stain and observed under a high-power light microscope to evaluate skin integrity by following the published protocol of Garg et al. (2016c), and the skin integrity of the Mkt-gel treated and untreated control animals compared.

### Stability of NLC and NLC-Gel Formulations

Physical and chemical stability of NLCs and NLC gel involved in short-term observations of different characteristic features were studied in accordance with the findings of Garg et al. (2016c). Possible changes in physical appearance, such as decoloration, gel consistency, and odor and appearance of drug crystals or precipitates were the stability parameters studied. The prepared formulations were studied at different temperature conditions (RF; 5 ± 3°C), room temperature (RT; 25 ± 2°C/60 ± 5% RH), and elevated temperature (HT; 40 ± 2°C/75 ± 5% RH) for over a period of 3 months (Garg et al., 2016c) as per ICH guidelines Q1A(R2) (Sharma et al., 2016b).

### Statistical Data Analyses

Data are shown as mean ± SD, and statistical analyses were carried out using one-way analysis of variance. The Tukey–Kramer multiple comparison post-hoc test using GraphPad InStat™ software (GraphPad Software Inc., CA, United States) was performed. The statistical differences are represented as *p < 0.05, **p < 0.01, ***p < 0.001, and ns = not significant (*p* > 0.05).

## Results and Discussion

### Solubility Studies

Solubility of the drug in various lipid oils, lipids, and surfactants was assessed, and Transcutol and Labrasol were shown to facilitate maximum solubility of ACE among a screened list of oils (Supplementary Table S1). Greater solubility of ACE was seen with cetyl alcohol and Compritol. PEG-300 and PEG-200 showed good solubilizing potential among the surfactants, but tween brought about moderate solubility of ACE. Furthermore, methanol and ethanol: water mixture proved to be better cosurfactants for drug solubility than isopropanol, ethanol, and absolute ethanol. As shown by Müller et al. (2007) and Garg et al. (2016b, 2016c), the drug was incorporated in the lipid matrix, dissolved or dispersed in lipid nanoparticles. Therefore, drug solubility in the lipid matrix is an important limiting factor for controlled drug release from prepared NLCs. Hence, Transcutol, PL S-100 and ethanol, and SA/GMS/CA were the finalized lipid oil, cosurfactant, and solid lipid, respectively, for the NLC formulations.

### Phase Diagram

**FIGURE 1:**
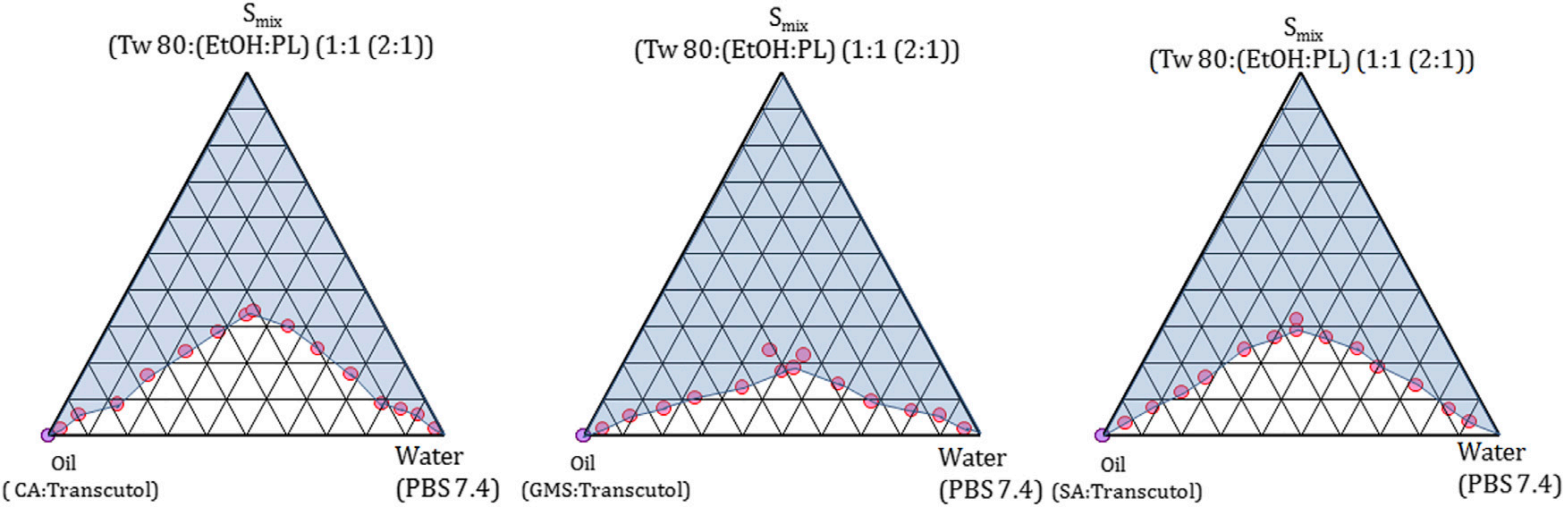
Phase diagram of NLCs prepared using different lipids.

**SCHEME 1:**
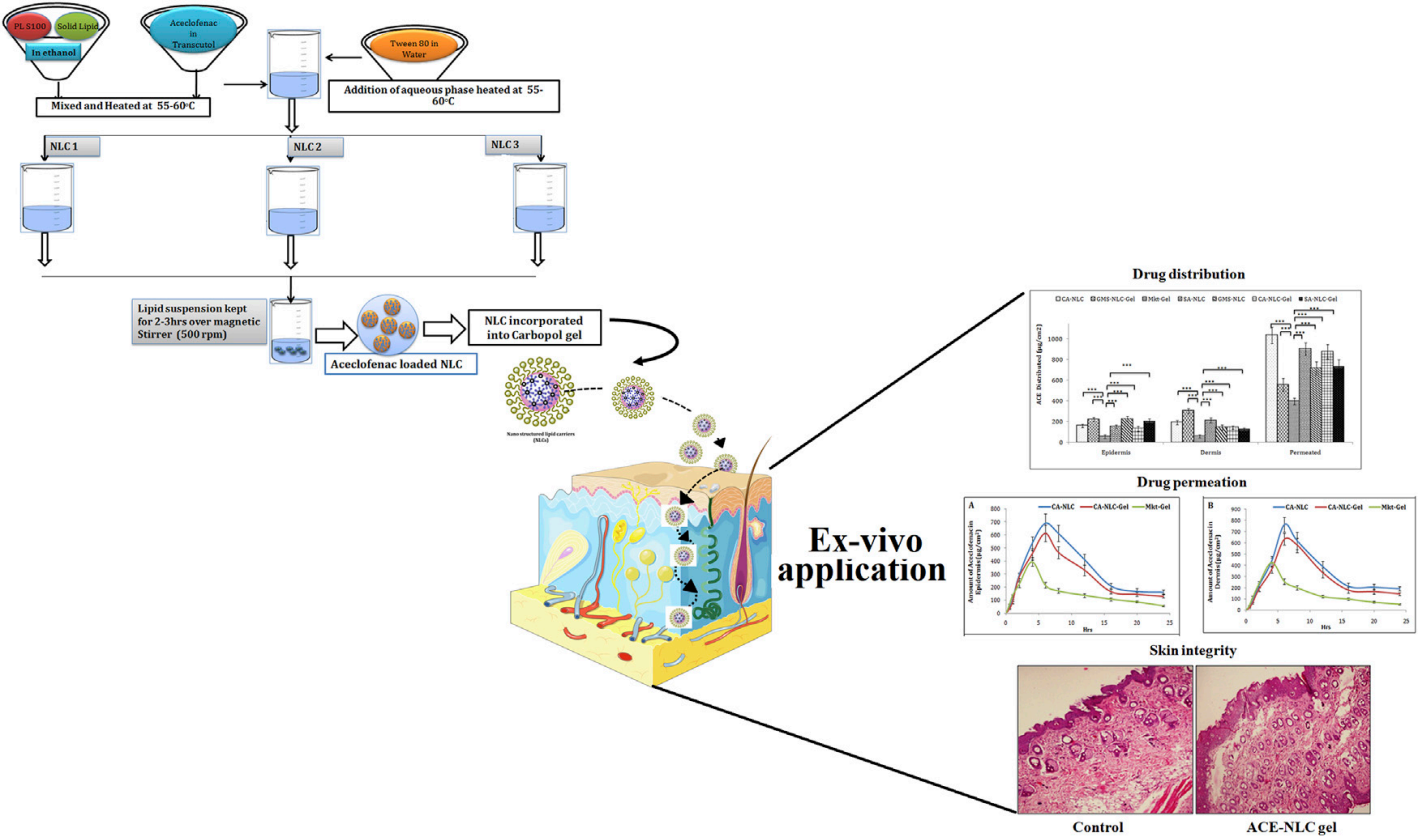
Schematic presentation of preparation of NLCs using three different techniques to establish ACE-NLC-gel formulation useful for transdermal applications for developing interventional strategies against inflammatory disorders.

Raw materials were chosen based on their solubility properties to prepare the ME region. A probable ME region was explored using pseudo phase diagrams (Figure 1) (Supplementary Table S2), prepared by the titration method (Aboofazeli et al., 1994; Aboofazeli et al., 1995; Garg et al., 2016b; Garg et al., 2016c), in branched series with serial changes in composition (Scheme 1). Phase boundary points were obtained and presented using Gibbs phase triangle, showing changes in phase behavior as proportionate to the changes seen in weight fractions of the water–oil–surfactant mixture during titration. In accordance with the findings of Garg et al. (2017), each apex of the triangle represents 100% of a single phase, the opposite side of the triangle represents 0% of a particular phase (Figure 1), and each axis of the triangle represents one of the three binary mixtures: aqueous–surfactant, aqueous–oil, and surfactant–oil.

The changes in the ratio of the surfactant: cosurfactant and ethanol: phospholipid (changes in the internal ratio of the cosurfactant) were proportional to the ratio of S_mix_ with the aqueous and oil phases (Garg et al., 2017). Ethanol influences the ME region when the internal ratio of ethanol with respect to PL increases, but when the ME region showed an increase beyond an extent, it scarcely left an effect on the ME region. Therefore, cosurfactants (ethanol and PL) were chosen in the ratio 2:1, and the ME region showed an increase up to the extent of the increased surfactant to cosurfactant ratio. The ratio of the surfactant to cosurfactant was kept in equal proportion leading to a sizeable ME region with GMS when compared to SA and CA (Figure 1), as was reported by Garg et al. (2017). The composition and weight ratio of different components of the phase diagram are summarized in Supplementary Table S2.

### Formulation and Characterization of Drug-Loaded NLCs and NLC Gel

NLCs were formulated by a novel approach to avoid degradation of ACE due to the extreme temperature conditions of conventional preparation methods (Ganesan and Narayanasamy, 2017; Ghasemiyeh and Mohammadi-Samani, 2018) (Scheme 1). The prepared NLCs were further incorporated in Carbopol 940 gel and characterized for particle size, PDI, zeta potential, and drug encapsulation efficiency (Table 1). Similarly, NLC gel was characterized with respect to the rheological characteristic and texture profile.

**TABLE 1:** Characterization of NLC formulations with respect to size, zeta potential, and EE.

Sr.No.	Solid lipids or Transcutol	Method used	Zeta potential	Particle size	PDI	EE (%)
1	GMS	Homogenization (NLC-1)	−14.78 ± 2.1	230 ± 10.4	0.254	70.28 ± 2.4
2	SA	Homogenization (NLC-1)	−13.38 ± 1.1	161 ± 8.9	0.211	75.8 ± 2.3
3	CA	Homogenization (NLC-1)	−14.2 ± 0.9	205 ± 9.8	0.190	65.54 ± 1.9
4	GMS	Probe sonication (NLC-2)	−16.8 ± 2.2	342 ± 13.4	0.311	66.34 ± 1.5
5	SA	Probe sonication (NLC-2)	−13.9 ± 1.7	351 ± 17.8	0.281	71.20 ± 2.1
6	CA	Probe sonication (NLC-2)	−12.9 ± 0.8	311 ± 15.4	0.275	62.29 ± 1.7
7	GMS	Sonication with homogenization (NLC-3)	−15.42 ± 2.8	219.1 ± 9.2	0.217	74.90 ± 2.2
8	SA	Sonication with homogenization (NLC-3)	−13.58 ± 1.8	151.5 ± 11.5	0.218	69.90 ± 3.2
9	CA	Sonication with homogenization (NLC-3)	−**11.35 ± 1.3**	**187.5 ± 5.2**	**0.233**	**80.90 ± 2.6**

**FIGURE 2:**
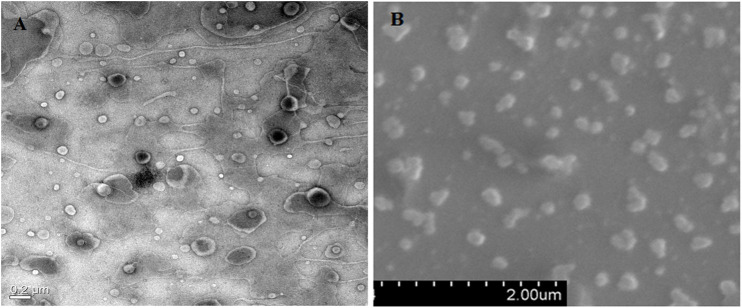
Microscopic analysis of shape and size of prepared formulations. **(A)** HR-TEM analysis and **(B)** SEM analysis of ACE-loaded CA-NLCs.

Differences in particle size and the PDI of the prepared NLC formulation were observed when prepared using three techniques. NLCs were prepared by homogenization (NLC-1) and probe sonication with homogenization (NLC-3) and had a more uniform size distribution with small size than did the NLCs that were prepared by the probe sonication method (NLC-2). High mechanical stress and time are required to produce fine-sized particles; therefore, most of the NLCs were seen in the size range 150–400 nm (Garg et al., 2016c). The PDI values of NLCs estimated to be in the range 0.19–0.25 suggest uniform size distribution of the prepared formulations. The polydispersity parameter showing a value less than 0.25 confirmed the homogeneous nature of the prepared nanoparticles, leading to minimal chances of predisposition to the aggregation (Mitri et al., 2011; Yang et al., 2019). Furthermore, NLC stability is directly proportional to the acceptable range of zeta potential values from −12.0 to −16.0. The negative charge on NLCs shows repulsion and therefore avoids particulate aggregation, thereby conferring stability to the prepared formulations (Jain et al., 2003). Characterization of the formulations with respect to the surface morphology of the optimized NLC (CA-NLC) formulation was carried out using TEM and SEM analyses (Figures 2A,B). Formulated particles were spherical and nanometric in size with narrow size distributions, and the size of the smooth round edged nanoparticles was below 200 nm (Figure 2A).

Drug encapsulation efficiency of the NLC formulation was calculated using different techniques (Table 1). Entrapment efficiencies of all NLC formulations were determined from 60 to 80%, but the highest encapsulation was found with NLC-3 (80.90 ± 2.6) formulation prepared by sonication and homogenization method using CA-lipid. The higher EE of NLC gel suggests higher solubility of ACE in the selected lipid matrix (Khurana et al., 2014; Garg et al., 2017; Haider et al., 2020). Furthermore, lower homogenization speed and short sonication period and structural features, wherein the use of solid lipid and liquid lipid jointly leads to an unorganized lipid matrix, are capable of accommodating greater drug quantity (Khurana et al., 2013). The final formulations were opted on the basis of their EE, size of formulated particles, and PDI. Higher EE, small size, and lower PDI led to the selection of NLC-3 for preparing NLC gel. Moreover, as the drug has to breach the polymeric and lipid matrix, formulations with a higher drug payload were selected and preferred.

NLC-3 formulations were incorporated into Carbopol 940. Carbopol (a commonly used acrylic acid polymer) may be crosslinked with poly-alkenyl ethers or divinyl glycol, and as reported by Chawla and Saraf (2012), it readily absorbs water, gets hydrated, and swells. Different grades of Carbopol polymers exhibit different rheological properties, depending on their particle size, molecular weight between cross-links (Mc), distributions of Mc, and the fraction of total units appearing as terminal units, i.e., free chain ends. The Mc for Carbopol 940 has been reported to be 1,450 monomer units (or 1,450 × 72 = 104,400 g/mol). Consistent with the findings of Chawla and Saraf (2012) and Raj et al. (2016), Carbopols were found to be nontoxic and nonirritant materials without having any allergic reactions in humans when applied topically (Das et al., 2013).

Textural properties (firmness, spreadability, and stickiness) of topical formulations are important for patient acceptability. Texture profiling in terms of measurement of force and work was carried out using male cones for penetration and detachment from the test formulation of female cones. The force of penetration indicates to the gel strength of a formulation, whereas the force of adhesion denotes to extrusion force (force required to remove the gel from the containers; tube). Work of shear and work of adhesion exhibit spreadability and stickiness characteristics of the gel. These properties were determined using TTC Spreadability Rig fitted on Texture Analyzer, and the gel was found to be good and smooth in appearance (Supplementary Figure S1).

**FIGURE 3:**
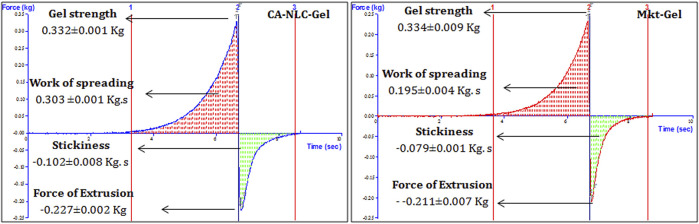
Comparative TPA of CA-NLC-gel and Mkt-gel formulations.

**FIGURE 4:**
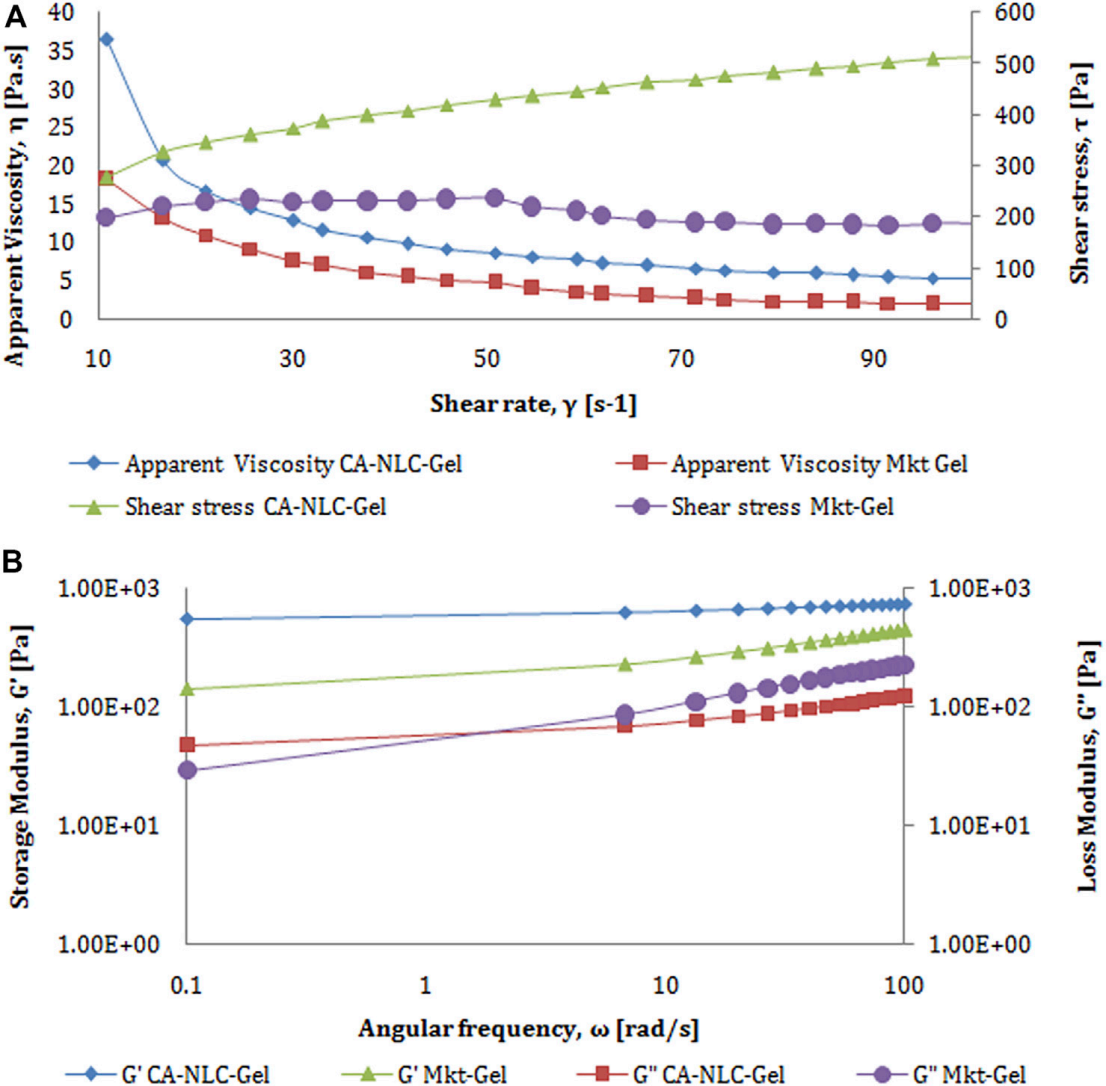
Characterization of rheology behavior of developed CA-NLC-gel and Mkt-gel formulations with respect to **(A)** shear rate *vs.* apparent viscosity and **(B)** angular frequency *vs.* storage modulus.

Peak force was shown as a measurement of gel strength for TPA (Figure 3). Therefore, peak force is directly related to the strength of gel network, and the area of curve up to this point was taken as the measurement of work of shear, which reflects spreadability of the samples (Figure 3). As a consequence of the negative region of the graph, the results obtained with the samples were seen lifted primarily on the upper surface of the male cone on return. Garg et al. (2017) reported that the negative region is due to back movement indicating adhesion or resistance to flow out of the disk. The maximum negative value is the force of adhesion required for the gel to extrude it from the tube. Furthermore, the area of the negative region of the curve was taken as work of adhesion or stickiness (Garg et al., 2017). The force of extrusion of all developed formulations and Mkt-gel was found to be nearly the same, thus the results of Garg et al. (2016c) were also validated by the present data, wherein better spreadability and gel strength of the developed formulations than the Mkt-gel was seen. Rheological study shows the flow behavior of NLC gel. Furthermore, as reported earlier, the Herschel–Bulky model was fitted for NLC gel, as it was suitable for this study. Rheograms represent the values of viscosity (Y_1_) and shear stress (Y_2_) obtained at varying shear rates (X1) (Figure 4A). Data obtained from this study suggest a correlation between decreased viscosity and increased shear stress. Viscosity decreased up to 3.7 from 20 Pa^.^s with respect to the shear rate from 10 to 100/sec for CA-NLC-gel. Although viscosity showed a decrease with increased shear stress, a decrease in viscosity was observed with decreased shear rate for the Mkt-gel formulation at 100/sec shear rate (Garg et al., 2016c; Garg et al., 2017). The trend between viscosity and shear rate was similar to that found for Mkt-gel and CA-NLC formulations. By contrast, shear stress was directly proportional to shear rate, and shear stress showed an initial sharp increase followed by a steady increase in terms of shear rate reaching up to 378 Pa for CA-NLC-gel formulation. In contrast, the Mkt-gel formulation showed an initial increase in shear stress (upto 235 Pa at the mid of shear rate) and then a gradual decrease reaching 185 Pa by the end point giving a curve-like graph for shear stress. NLC gel displayed pseudoplastic flow from a colloidal network structure and therefore adjusted itself in the direction of flow. Additionally, thixotropy, a desirable feature for transdermal applications, was observed in NLC gel (Lee et al., 2009; Han et al., 2012; Ghica et al., 2016; Vigani et al., 2020).

The results obtained with amplitude LVR test showed that storage modulus (G′) is higher than loss modulus (G″) assuring of greater gel elasticity, which in turn, is responsible for lesser dissipation of energy. The loss in modulus can be higher only when the sample is predominantly viscous. The graph plotted between G′, G″ at Y_1_, shear stress at Y_2_, and strain at X_1_ was found to be linear (Figure 4B). G′, G″, and shear stress of CA-NLC-gel are seen to be significantly higher than for Mkt-gel formulation: 58.5, 93.1, and 110 Pa for Mkt-gel and 154, 122, and 197 Pa for CA-NLC-gel at 100% strain, respectively. Furthermore, loss in tangent is the measurement of energy loss to stored energy in cyclic deformation (tan *δ* = *G*″/*G*′), and the value of tan *δ* < 1 is an indicator of prevalent elastic demeanor (Negi et al., 2014; Garg et al., 2016c).

The outcome of the frequency sweep study made it possible to determine the internal changes in gel structure. A test was conducted at 1% system at an extended frequency sweep (100–0.1 Hz) at room temperature (25 ± 0.5°C). The values for storage modulus (G′), loss modulus (G″), and complex viscosity (η*) were laid down across the frequency range. Consistent with the findings of Jain et al. (2014), no crossover was seen at room temperature (Figure 4) in this study.

G′, G″, and complex viscosity of the Mkt-gel were estimated to be lower than were for CA-NLC-gel; these were 232 Pa, 86.3 Pa, and 36.7 Pa.s for Mkt-gel and 436 Pa, 72.6 Pa, and 65.4 Pa.s for CA-NLC-gel, respectively. Furthermore, storage and loss modulus showed an increase with decrease in complex viscosity in the linear mode. This pattern delineates the higher efficiency of the sample, i.e., increased modulus with decreased measurement time (frequency = 1/time). Briefly, at a particular phase and viscosity, the formulation could be easily transported and stored at ambient temperature, provided it was not subjected to any shear changes that may alter its viscosity and structural stability (Garg et al., 2016c; Garg et al., 2017). The texture profile and rheogram of the NLC-based formulation showed a nearly similar pattern, therefore only CA-NLC-gel data have been presented here.

### *In Vitro* Release Study

**FIGURE 5:**
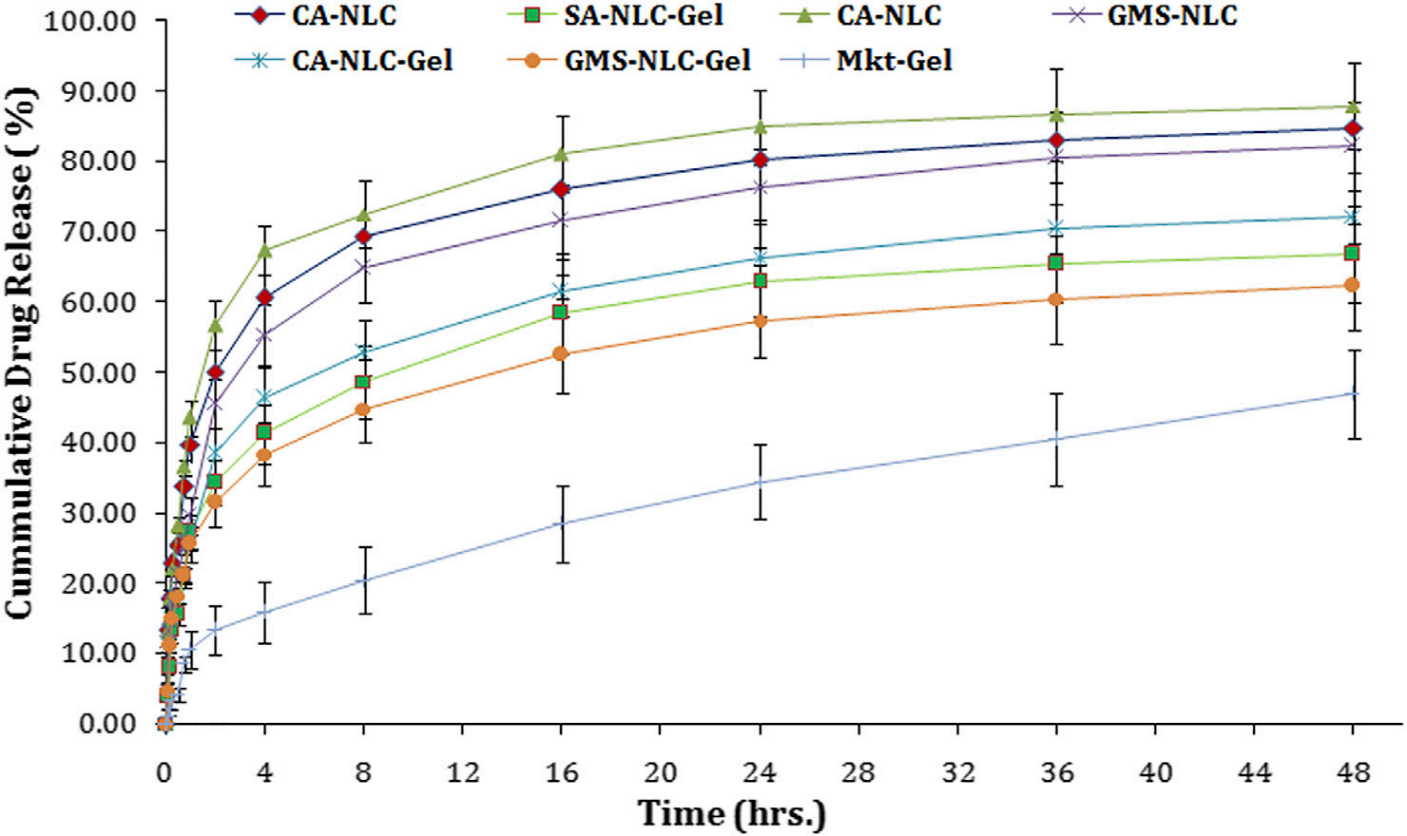
Determination of *in vitro* cumulative drug release using various NLC formulations tested (CA-NLC, SA-NLC, SA-NLC-gel, GMS-NLC, CA-NLC-gel, GMS-NLC-gel, and Mkt-gel).

CA-NLCs, SA-NLCs, and GMS-NLCs followed a biphasic release pattern of drug for 48 h. The initial release of 67.34 ± 4.45%, 60.56 ± 3.67%, and 55.38 ± 4.78% from CA-NLCs, SA-NLCs, and GMS-NLCs, respectively, was observed in 4 h and then sustained release was seen for up to 48 h (Figure 5). 87.83 ± 5.45%, 84.48 ± 6.45%, and 82.28 ± 5.45% release was calculated at the end of the 48^th^ hour for CA, SA, and GMS-NLCs, respectively. A significantly higher (*p* < 0.01) release of the drug from NLCs was observed at the 48^th^-hour time point than was observed for the NLC-gel formulation. Cooling from a higher temperature to room temperature favors augmentation and enrichment of drugs in the outer layers of formulated NLCs particles that show superficial entrapment, thus causing initial burst release (Zur Mühlen et al., 1998; Khosa et al., 2018; Siahdasht et al., 2020). This release is explained due to the rapid release of traces of bound/adsorbed drug on the surface or by the presence of the drug just underneath the stratum of NLCs. The sustained release pattern of ACE observed during the period of 48 h could be attributed to the diffusion of the drug through the lipid matrix of the NLCs or a slow degradation of the lipid matrix in the release medium.

Suboptimal drug release was seen with the NLC-gel formulation when compared with that seen with the NLC formulation. This decrease in release is probably due to hindrance created by the gel matrix to the NLC formulation. The drug release was 46.38, 41.26, 38.38, and 15.78% at the end of the 4^th^ hour and 72.31, 66.28, 62.3 5, and 46.95% at the end of the 48^th^ hour for CA-NLC-gel, SA-NLC-gel, GMS-NLC-gel, and Mkt-gel, respectively. Furthermore, NLC-based gel formulations were shown to have a higher *in vitro* release profile than did the Mkt-gel. Furthermore, the drug release from the CA-NLC-gel formulation was highly significant (*p* < 0.001) when compared with the Mkt-gel formulation after 24 h.

Percent drug release was found lesser in GMS-NLC and GMS-NLC-gel than in other lipids formulations. Restricted free drug movement was the primary reason for the suboptimal release with GMS. The compact structure of the GMS matrix allowing liquid flow, its internal molecular organization, and the size of the network mesh can all affect drug release dissolved in liquid phase, as it hinders free diffusion (Huttenrauch and Fricke, 1979).

Several kinetic models describe drug release from immediate and modified release dosage forms. The model that best fits the release data was assessed using correlation coefficient (r) value, which is used as the criteria to choose the best model to understand and describe drug release from NLCs and NLC-based polymeric gel formulations. The r^2^ values for the different kinetic models are summarized in Table 2. The release data from NLCs and NLC-gel formulations were best fitted into the Korsmeyer–Peppas equation for release kinetics with Fickian diffusion. The Mkt-gel formulation was best fitted into the Higuchi equation as shown in Table 2. The n values for developed NLCs and NLC-gel formulations were found to be between 0.1304 and 0.4406, and these values show that formulations follow the Korsmeyer–Peppas equation and exhibit Fickian diffusion. Moreover, the mechanism of drug release follows drug diffusion through the lipid matrix (Jana et al., 2014).

**TABLE 2:** Drug release models for the *in vitro* release study.

Formulation	Drug release models (^R2^)				
	Zero order	First order	Higuchi	Hixon–Crowell	Korsmeyer–Peppas
CA-NLC	0.6113	0.4573	0.8117	0.7337	0.8677
CA-NLC-gel	0.768	0.386	0.925	0.845	0.936
SA-NLC	0.6544	0.4981	0.8478	0.7619	0.949
SA-NLC-gel	0.6501	0.4217	0.8432	0.7186	0.8939
GMS-NLC	0.6875	0.5313	0.8736	0.784	0.962
GMS-NLC-gel	0.7031	0.4658	0.8829	0.7629	0.9109
Mkt-gel	0.9073	0.5586	0.9887	0.936	0.9608

### *Ex-Vivo* Study of Skin Permeation

**FIGURE 6:**
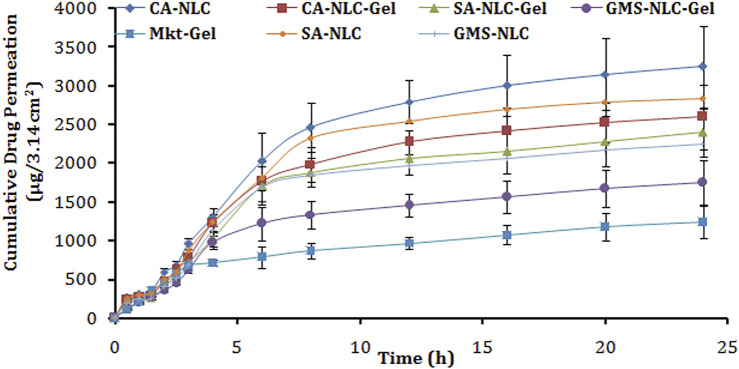
Cumulative drug permeation/e*x vivo* skin permeation analysis of different gel formulations tested (CA-NLC, SA-NLC, SA-NLC-gel, GMS-NLC, CA-NLC-gel, GMS-NLC-gel, and Mkt-gel).

The ear pinnae of the pig closely matches the human skin and hence was used to determine drug permeation across the skin (Touitou et al., 1998) for structural and barrier characteristics (Neubert and Wohlrab, 1990; Abd et al., 2016; Neupane et al., 2020). The results of this study showed that greater drug quantity permeated across the skin layers with the developed formulation than with the conventional Mkt-gel formulation (Figure 6; Table 3). The highest cumulative drug permeation (>3200 µg) was seen with CA-NLC when compared with that from the CA-NLC-gel (∼2600 µg) in 24 h (Q_24_) and Mkt-gel (1000 µg) formulations (Table 3). The data from this study suggest that the NLC formulation showed significantly higher permeation than the NLC-incorporated gel formulation and that more than 90% of the drug was recovered during *ex vivo* studies, confirming the effective permeation and retention of the drug.

**TABLE 3:** Characteristic skin transport parameters from various ACE formulations.

Formulation code	Cumulative drug permeated in 24 h (Q_24_) (µg)	Permeability coefficient (P) (cm^−2^.h^−1^)	Steady state permeation flux (jss) (µg/cm^2^ h)	ER
**CA-NLC**	**3250.46 ± 18.73**	**2.10E-02**	**118.41**	**1.59**
SA-NLC	2840.14 ± 17.92	1.75E-02	98.92	1.33
GMS-NLC	2250.83 ± 15.31	1.86E-02	104.90	1.41
**CA-NLC-gel**	**2600.52 ± 19.28**	**1.89E-02**	**106.43**	**1.43**
SA-NLC-gel	2400.17 ± 17.19	1.58E-02	88.98	1.2
GMS-NLC-gel	2750.15 ± 24.28	9.89E-03	55.76	0.75
Mkt-gel	1240.59 ± 12.28	1.31E-02	74.14	1

Steady-state permeation flux (Jss) (µg/cm^2^ h) of the drug was calculated from the slope of regression lines fitted to the linear portion of the permeability profiles as shown by the findings of Liu et al. (2000) and Garg et al. (2017). The cumulative amount of permeated drug (Q_24_), permeation rate, and permeability coefficient values of ACE released from NLC/NLC-gel formulations reached statistical significance (*p* < 0.001). The value of Jss was the highest for CA-NLC followed by the CA-NLC-gel formulation and was estimated to be the lowest for GMS-NLC-gel. The magnitudes of flux of the drug from NLCs and NLC gel were two fold higher than for the Mkt-gel formulation.

The percentage of drug permeated from each formulation (NLC gels and Mkt-gel) after 24 h was calculated to determine the ER (Table 3). The ER supports significant (*p* < 0.01) permeation profiling of CA-NLC and CA-NLC-gel formulations than it does for SA-NLC and SA-NLC–based gel formulations, when compared with Mkt-gel and GMS-NLC-gel formulations. Increased drug permeation was due to enhanced permeation and retention of drug, as NLCs play a crucial role to change the cellular and physiological ambience (Garg et al., 2017). The enhancement effect of the gel can be correlated to hydrogel and its positive aspects, to render additional advantages in a hydrating atmosphere to NLCs (Garg et al., 2016c). Thus, prepared formulations effectively make drug molecules accessible within the skin layers and retain the drug at the target site (Garg et al., 2016c; Sabzichi et al., 2016; Yu G. et al., 2021). A greater permeation profile of the NLC formulation can be described on the basis of the carrier effect and its permeation-enhancing ability when delivered *via* the topical route. Particle size plays an important role during transdermal administration, as small-sized nanoparticles easily penetrate the stratum corneum following intracellular pathways (Ghosh and Michniak-Kohn, 2012). The nano size of the NLCs help maintain close proximity with the corneocytes of the stratum corneum to retain and store drug in the skin. The negatively charged particles with negative zeta potential enhance the permeation of nanocarriers across the skin layers (Kohli and Alpar, 2004; Garg et al., 2016c). Furthermore, the higher drug EE of NLCs exhibits enhanced fluidity of the drug and synergism among ethanol, phospholipid, Transcutol, tween 80, poloxamer, solid lipids, and skin lipids, leading to increased flux (Dayan and Touitou, 2000). This, in turn, facilitates the percutaneous absorption of the drugs, and lower concentrations of PL molecules are incorporated in the skin layers and fluidized to loosen the lipid matrix of the skin, thereby promoting drug permeation (Yokomizo and Sagitani, 1996; Garg et al., 2016c). Tween 80 and poloxamer facilitate active transport of NLCs through the skin *via* various mechanisms, including endocytosis, and solubilization of endothelial cell membrane lipids (Jain et al., 2015; Garg et al., 2016c) fluidize the membrane resulting in enhanced drug permeability. The incorporation of NLCs into Carbopol gel decreases permeability of the drug probably due to the enhanced viscosity of the hydrogel system when compared with plain NLCs. The viscous gel retards mobility and hampers permeation of the drug (Peltola et al., 2003; Garg et al., 2016b; Garg et al., 2016c).

### Drug Distribution Across Skin Layers

**FIGURE 7:**
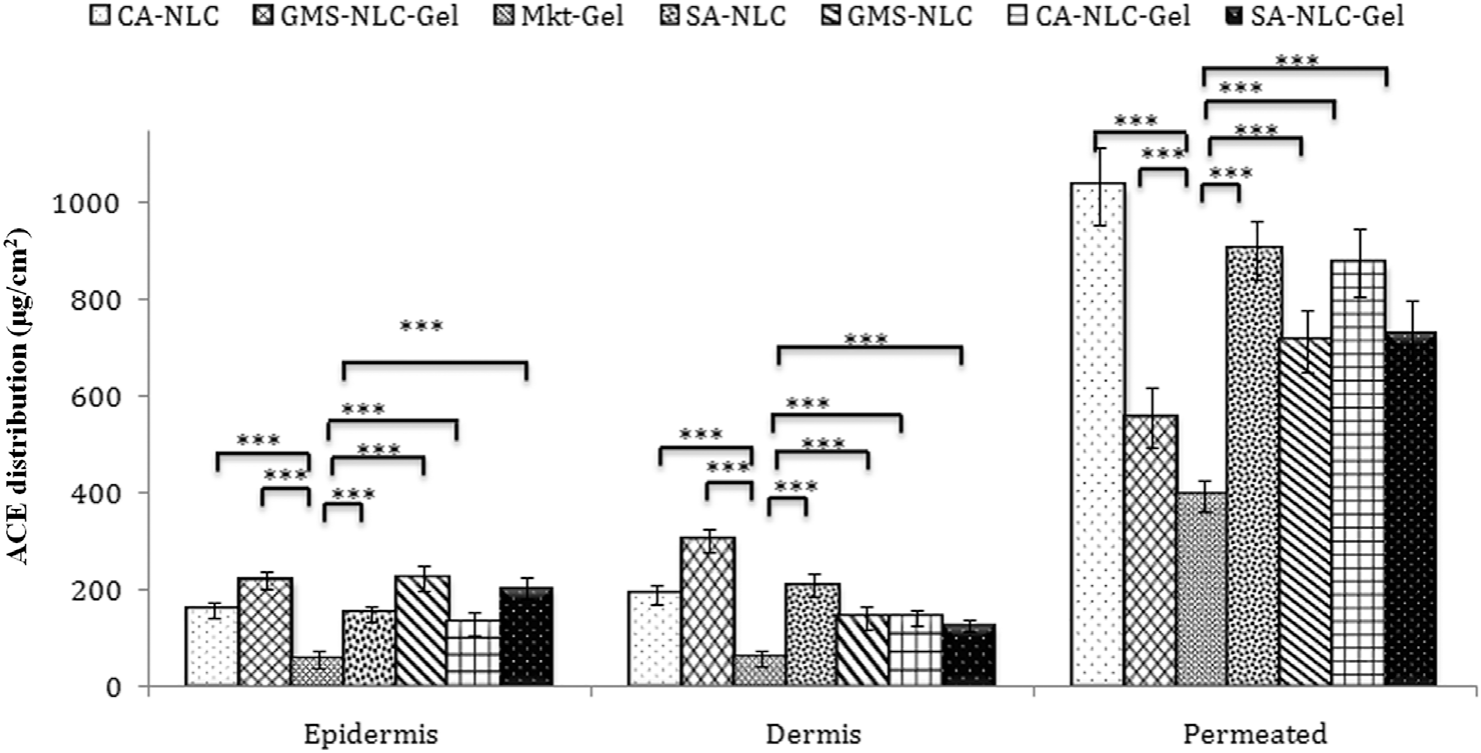
Assessment of ACE distribution across the skin layers (epidermis and dermis) and quantification of ACE present in the epidermis and dermis, to quantify the total drug permeated using different formulations (CA-NLC, SA-NLC, SA-NLC-gel, GMS-NLC, CA-NLC-gel, GMS-NLC-gel, and Mkt-gel). Statistical data are expressed as *p < 0.05 (significant), **p < 0.01 (moderate significant), and ***p < 0.001 (highly significant).

Skin deposition attributes of NLCs and NLC gel were designed and targeted to explore their potential in forming drug reservoirs deeper in the skin. The results of this study demonstrate the quantifiable amounts of drug deposition in the epidermis and dermis by the NLC formulations (Figure 7). Sizeable quantity of the drug was also detected in the receptor. Hence, it is inferred that the drug penetrated through the skin barrier to dermal vasculature.

Different NLC-gel formulations showed varying degrees of drug accumulation in the different compartments, but drug concentration in the dermis was seen to be significantly (*p* < 0.001) higher than in the epidermis (Figure 7); the Mkt-gel formulation behaved otherwise. The data from this study showed higher drug concentrations in different compartments with the developed formulations than the Mkt-gel formulation. Therefore, it is inferred that skin permeation of NLC-based gel formulation is higher (*p* < 0.001) than is for Mkt-gel formulation. Nearly threefold drug retention in the skin was observed with the developed formulation when compared to the Mkt-gel formulation. The authors postulate that this is due to the formation of microreservoirs of drug molecules within the dermis layers which is because of the integration of phospholipids and skin lipids (Raza et al., 2013; Garg et al., 2016c).

Dermal retention of the drug was attributed to increased contact with the skin layers, the occlusive effect, and sustained release due to the properties of NLCs. Small-sized NLCs improve skin penetration ability of nanoparticles and enhance lipophilicity of the dermal layer, circumscribing the valuable partitioning of hydrophobic drugs. Therefore, drug penetration of the skin layers may be easily achieved by using NLCs for the delivery of drugs. Drug was entrapped in the lipid matrix and later incorporated in Carbopol 940 gel adhered to the skin. This nanocarrier arrangement increased contact time of the formulation at the application site. Skin distribution data of this study show advancement of NLC-based drug delivery and their use as a versatile topical delivery system with minimal transdermal localization and higher skin penetration. *In vitro* release and *ex vivo* skin permeation studies advocate NLC-based formulations as “prominent transdermal delivery systems,” and the CA-NLC–based formulation was found to be the best among all the preparations tested (Garg et al., 2016c).

### Dermatokinetic Modeling

**FIGURE 8:**
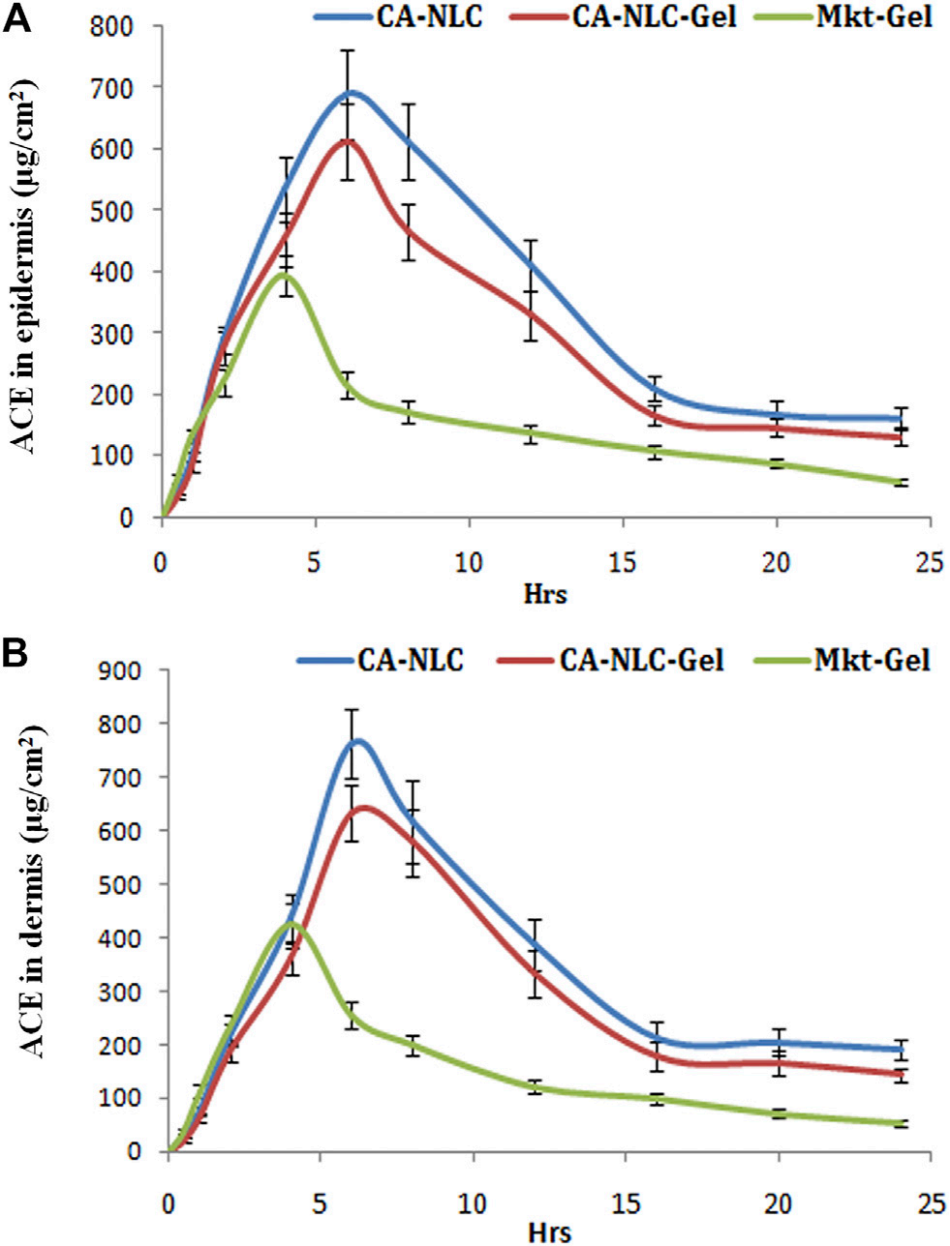
Quantification of ACE in the **(A)** epidermis and **(B)** dermis (ug/cm^2^) with CA-NLCs, CA-NLC-gel, and Mkt-gel.

The authors quantified distribution of ACE in the epidermis and dermis of the ear pinnae of the pig (Figure 8). Availability of ACE in the skin followed the one-compartment open body model (1CBM). The obtained data were processed using dermatokinetic modeling for 1CMB to reach an inference. The amount of ACE in the skin layers delivered by NLCs was higher (*p* < 0.001) than was for the Mkt-gel formulation (Table 4). All dermatokinetic parameters (AUC_0-24hrs_, $C_{Max}^{skin}$ , $T_{Max}^{skin}$ , K_e_, K_p_) of both the delivery systems (CA-NLC and CA-NLC-gel) were found to be highly significant (*p* < 0.001) when compared with those of Mkt-gel formulation in the dermis (Figure 8; Table 4). The data from this study suggest that ACE released from both formulations showed maximum concentrations of 600–700 and 650–750 µg in the epidermis and dermis in 4–6 h, respectively, followed by an equilibrium stage in 3–12 h. Decreased levels of ACE were found to have permeated across the skin layers until a plateau had been achieved. This is probably due to the long-lasting and controlled transport of ACE by NLCs across the skin layers (Garg et al., 2016c). Drug permeation and elimination rates *via* the lipid matrix were found to be nearly the same for all formulations in this study. However, the elimination rate was more than the permeation rate for Mkt-gel formulation. This is explained on the basis of lesser quantity of drug being permeated across the skin barriers than from rapid elimination of free ACE. These findings suggest that NLCs have improved the skin transportation characteristics of ACE by their design and composition when compared with the Mkt-gel formulation. This can be attributed to the favorable interaction between lipid/phospholipids of NLCs and skin lipids that may have rendered a synergist effect on the facilitation and interaction of ACE. In the end, significant quantities of the drug were found to have permeated, equilibrated, and been transported across the skin layers. These nanoscale drug carriers transport ACE through the skin layers quite efficiently and facilitate adequate drug supply to the diseased site (Garg et al., 2017).

**TABLE 4:** Various dermatokinetic parameters (mean ± SD) of ACE topical formulations in the epidermis and dermis (*n* = 6).

Dermatokinetics parameter	CA-NLC		CA-NLC-gel		Mkt-gel	
	Epidermis	Dermis	Epidermis	Dermis	Epidermis	Dermis
AUC_0-24hrs_ (µg/cm^2^/h)	9625.54 ± 946.91	10038.28 ± 927.50	7833.97 ± 805.42	8602.11 ± 293.10	3991.66 ± 330.83	3818.11 ± 365.73
$C_{\mathrm{Max}}^{\mathrm{skin}}$ (µg/cm^2^)	574.81 ± 43.89	551.67 ± 62.21	482.03 ± 38.52	476.67 ± 49.59	279.76 ± 28.23	305.86 ± 30.62
$T_{\mathrm{Max}}^{\mathrm{skin}}$ (h)	6.11 ± 0.63	6.69 ± 1.18	5.97 ± 0.84	6.63 ± 1.34	3.91 ± 0.76	4.18 ± 0.75
K_*p*_ (h^−1^)	4.26 ± 0.479	4.61 ± 1.03	4.08 ± 0.39	4.51 ± 0.04	1.37 ± 0.65	1.94 ± 0.36
K_*e*_ (h^−1^)	4.27 ± 0.89	4.66 ± 0.43	4.20 ± 0.37	4.68 ± 0.44	2.52 ± 0.061	2.60 ± 0.89
Cl (µg/h)	0.58 ± 0.08	0.56 ± 0.13	0.71 ± 0.11	0.65 ± 0.05	1.41 ± 0.025	1.47 ± 0.089

### Histopathology to Evaluate Cell Infiltration and Skin Integrity

**FIGURE 9:**
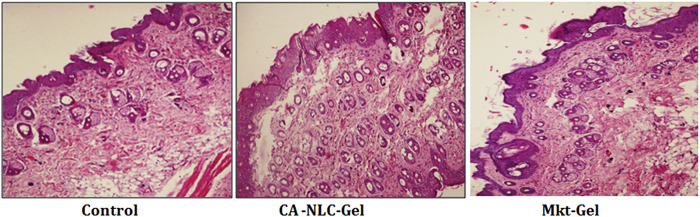
Evaluation of the skin integrity when the skin of pig ear pinnae was treated with CA-NLC-gel and Mkt-gel, and of the untreated control, using histopathogy (hematoxylin and eosin staining). Photomicrogaphs were taken at ×200 magnification.

The skin integrity of pig ear pinnae was assessed in this study using CA-NLC-gel, Mkt-gel, and untreated control (Figure 9). The skin was found to show normal keratinization and nonspecific changes, such as the presence of a moderate number of mast cells in the skin treated with the Mkt-gel formulation. However, CA-NLC-treated skin showed changes in the skin layers, with increased width of cell gaps and elongation of epidermal and dermal skin additions. Similarly, skin sections treated with CA-NLC-gel formulation showed normal keratinization, but inflammatory cell responses were not seen in the epidermal/dermal regions alongside the minimal changes observed in skin histology (Figure 9). This is attributed to the indirect contact of ME components with the skin due to the formation of gel. Finally, no significant change in skin integrity was observed in the *ex vivo* permeation study when the drug formulation was topically applied for 24 h; therefore, topical delivery of ACE-laden CA-NLC-gel systems might be safe to be licensed for clinics.

## Conclusion and Future Perspective

ACE-NLC hydrogel was successfully formulated, characterized, evaluated, and compared with the Mkt-gel formulation, *viz.*, *in vitro* release, texture, and rheology behavior; skin permeation and drug distribution; and dermatokinetic modeling, skin integrity, and stability. The developed NLC formulation (CA-NLC) showed fair particle size distribution in the size range 150–350 nm, elevated zeta potential levels (>−10 mV), low PDI (<0.2), and higher encapsulation efficiency (>75%) to maintain the spherical shape and smooth surface morphology. NLC-based formulations showed better cumulative drug permeation (Q_24_), permeation flux, and ER. Lipid nanocarriers (NLCs) facilitate drugs to accumulate and saturate in the epidermis and dermis, and to transport them across the skin layers for conferring therapeutic effect at diseased sites. The evaluation of *in vitro* skin permeation of ACE shows its capability to breach skin barrier by NLC-mediated ACE delivery. Indeed, these nanoscale drug carriers increase the permeation of ACE owing to their beneficial aspects during topical applications. Patient compliance and safety profiling confirmed by skin integrity studies establish CA-NLC and CA-NLC-gel formulations as a viable therapeutic option for inflammatory diseases such as RA. In essence, the findings of this study suggest the use of NLCs as a potential novel carrier for topical delivery of ACE to develop interventional therapeutic approaches for systemic inflammatory diseases. Additionally, the findings of Garg et al. (2016b, 2016c) on RA confirm the advantages of using NLCs over SLNs in terms of safety, bioavailability, biocompatibility, and help in modulating inflammation and inducing selective apoptosis. The data from this study suggest the use of NLCs as second-generation colloidal nanoparticles to develop a viable delivery option for ACE in treating systemic inflammatory diseases.

## Data Availability

The original contributions presented in the study are included in the article/Supplementary Material, and further inquiries can be directed to the corresponding author.

## Abbreviations

ACE, aceclofenac; NLCs, nanostructured lipid carriers; SLNs, solid lipid nanoparticles; NSAID, nonsteroidal anti-inflammatory drug; COX, cyclooxygenase; GMS, glyceryl monosterate; SA, stearic acid; CA, cetyl alcohol; HPLC, high-performance liquid chromatography; ME, microemulsion; PDI, polydispersity index; TEM, transmission electron microscopy; SEM, scanning electron microscopy; LVR, linear viscoelastic region; TPA, texture profile analysis; PBS, phosphate buffer saline; ER, enhancement ratio; 1CBM, one compartment open body model; RA, rheumatoid arthritis.
