# Corrigendum: Nanostructured lipid carrier–mediated transdermal delivery of aceclofenac hydrogel present an effective therapeutic approach for inflammatory diseases

**DOI:** 10.3389/fphar.2022.1002649

**Published:** 2022-08-25

**Authors:** Neeraj K. Garg, Nikunj Tandel, Sanjay Kumar Bhadada, Rajeev K. Tyagi

**Affiliations:** ^1^ University Institute of Pharmaceutical Sciences, Panjab University, Chandigarh, India; ^2^ Institute of Science, Nirma University, Ahmedabad, India; ^3^ Department of Endocrinology, Postgraduate Institute of Medical Education and Research (PGIMER), Chandigarh, India; ^4^ Division of Cell Biology and Immunology, Biomedical Parasitology and Nano-Immunology Lab, CSIR-Institute of Microbial Technology (IMTECH), Chandigarh, India

**Keywords:** aceclofenac, nanostructured lipid carrier, rheological behavior, texture profile, transdermal delivery

In the original article, there was an error in Figure 2 as published. By mistake, an incorrect image for Figure 2A was supplied. The corrected Figure 2 appears below.

**FIGURE 2:**
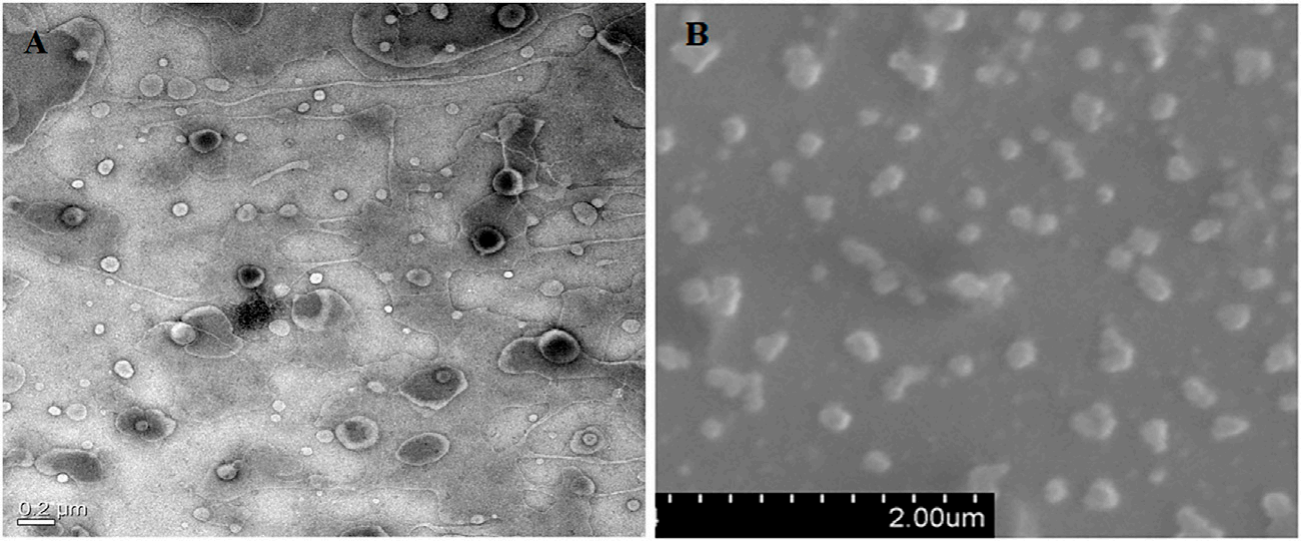
Microscopic analysis of shape and size of prepared formulations. **(A)** HR-TEM analysis and **(B)** SEM analysis of ACE-loaded CA-NLCs.

The authors apologize for this error and state that this does not change the scientific conclusions of the article in any way. The original article has been updated.

