# Supplementary material for: Nanostructured Lipid Carrier–Mediated Transdermal Delivery of Aceclofenac Hydrogel Present an Effective Therapeutic Approach for Inflammatory Diseases
Source: Front Pharmacol. 2021 Sep 20;12:713616. doi: 10.3389/fphar.2021.713616 (PMC8488093; doi:10.3389/fphar.2021.713616)
Supplement: Supplementary file 1 [file Table1.docx]

**SI Table 1. Solubility of aceclofenac in different oils, lipids, surfactants and co-surfactants**

| **Sr no** | **Solvents** | **mg/gm** |
| --- | --- | --- |
| **Solid Lipids** | | |
| 1 | Glyceryl monosterate | 145 ± 11.96 |
| 2 | Cetyl alcohol | 205 ± 13.26 |
| 3 | Capmul | 115 ± 10.56 |
| 4 | Stearic acid | 155 ± 12.48 |
| 5 | Cetylpalmitate | 145 ± 13.57 |
| 6 | Compritol | 198 ± 16.27 |
| **Lipid oils** | | |
| 1 | Transcutol | 514 ± 26.2 |
| 2 | Labrasol | 280 ± 11.34 |
| 3 | Labrofac | 95.89 ± 6.87 |
| 4 | IPM | 11.56 ± 1.12 |
| 5 | Olive Oil | 2.68 ± 0.12 |
| 6 | Ethyl Oleate | 6.21 ± 0.27 |
| 7 | Olic Acid | 8.22 ± 0.11 |
| **Surfactants** | | |
| 1 | Tween 20 | 153.22 ± 9.76 |
| 2 | Tween 40 | 142.52 ± 8.23 |
| 3 | Tween 80 | 147.95 ± 5.67 |
| 4 | PEG 200 | 272.80 ± 9.78 |
| 5 | PEG 300 | 308.82 ± 6.98 |
| 6 | PEG 400 | 306.17 ± 8.99 |
| 7 | Propylene Glycol | 21.77 ± 1.1 |
| 8 | Terpinol | 84.26 ± 3.6 |
| 9 | Lutrol L-44 | -- |
| **Cosurfactants** | | |
| 1 | Ethanol | 73.69 ± 6.87 |
| 3 | Ab ethanol | 56.85 ± 3.76 |
| 4 | IPA | 34.67 ± 2.98 |
| 5 | Methanol | 150 ± 11.32 |
| 9 | Ethanol: Water | 150 ± 8.34 |
| 10 | Methanol: Water | 130 ± 9.57 |
