# Supplementary figures and images for: Nanostructured Lipid Carrier–Mediated Transdermal Delivery of Aceclofenac Hydrogel Present an Effective Therapeutic Approach for Inflammatory Diseases
Source: Front Pharmacol. 2021 Sep 20;12:713616. doi: 10.3389/fphar.2021.713616 (PMC8488093; doi:10.3389/fphar.2021.713616)

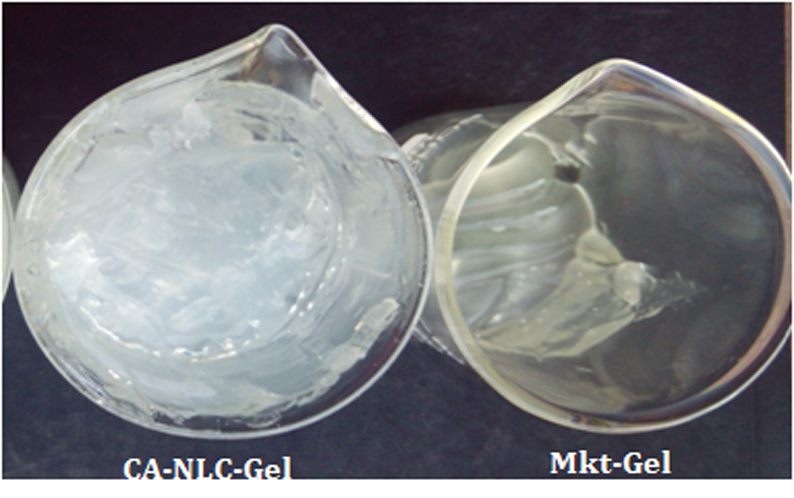

Supplement: Supplementary file 2 [file Image1.TIF]
