# Supplementary material for: Nanostructured Lipid Carrier–Mediated Transdermal Delivery of Aceclofenac Hydrogel Present an Effective Therapeutic Approach for Inflammatory Diseases
Source: Front Pharmacol. 2021 Sep 20;12:713616. doi: 10.3389/fphar.2021.713616 (PMC8488093; doi:10.3389/fphar.2021.713616)
Supplement: Supplementary file 3 [file Table2.DOCX]

**SI Table 2: Composition of different Pseudo-Ternary Phase Diagram**

| **S. No.** | **Oil Phase (8:2)** | | **Aqueous** | **Surfactant** | **Co-surfactant (w/w)** | | **Weight ratio of Surfactant: Co-surfactant (w/w)** |
| --- | --- | --- | --- | --- | --- | --- | --- |
|  | Solid Lipid | Lipid Oil |  |  | Ethanol | Phospholipid |  |
|  | SA | Transcutol | PBS (pH 7.4) | T80 | 2 | 1 | 1:1 |
|  | SA | Transcutol | PBS (pH 7.4) | T80 | 2 | 1 | 1:2 |
|  | SA | Transcutol | PBS (pH 7.4) | T80 | 2 | 1 | 2:1 |
|  | CA | Transcutol | PBS (pH 7.4) | T80 | 2 | 1 | 1:1 |
|  | CA | Transcutol | PBS (pH 7.4) | T80 | 2 | 1 | 1:2 |
|  | CA | Transcutol | PBS (pH 7.4) | T80 | 2 | 1 | 2:1 |
|  | GMS | Transcutol | PBS (pH 7.4) | T80 | 2 | 1 | 1:1 |
|  | GMS | Transcutol | PBS (pH 7.4) | T80 | 2 | 1 | 1:2 |
|  | GMS | Transcutol | PBS (7.4) | T80 | 2 | 1 | 2:1 |
